# BMSC-derived exosomes promote osteoporosis alleviation via M2 macrophage polarization

**DOI:** 10.1186/s10020-024-00904-w

**Published:** 2024-11-19

**Authors:** Yanbin Zhang, Jing Bai, Bin Xiao, Chunyan Li

**Affiliations:** 1 Department of Spine Surgery, National Center for Orthopaedics, Capital Medical University Affiliated Beijing Jishuitan Hospital, Beijing, 100035 People’s Republic of China; 2 Department of Trauma and Joint, The Third Affiliated Hospital of Beijing University of Traditional Chinese Medicine, Beijing, 100029 People’s Republic of China; 3 Department of Clinial Laboratory, Capital Medical University Affiliated Beijing Jishuitan Hospital, Xinjiekou No. 31 East Street, Xicheng District, Beijing, 100035 People’s Republic of China

**Keywords:** Exosome, Bone mesenchymal stem cells, Polarization, TRIM25, TREM1, Ubiquitination

## Abstract

Osteoporosis is characterized by reduced bone mass due to imbalanced bone metabolism. Exosomes derived from bone mesenchymal stem cells (BMSCs) have been shown to play roles in various diseases. This study aimed to clarify the regulatory function and molecular mechanism of BMSCs-derived exosomes in osteogenic differentiation and their potential therapeutic effects on osteoporosis. Exosomes were extracted from BMSCs. Bone marrow-derived macrophages (BMDMs) were cultured and internalized with BMSCs-derived exosomes. Real-time quantitative PCR was used to detect the expression of macrophage surface markers and tripartite motif (TRIM) family genes. BMDMs were co-cultured with human osteoblasts to assess osteogenic differentiation. Western blot was performed to analyze the ubiquitination of triggering receptor expressed on myeloid cell 1 (TREM1) mediated by TRIM25. An ovariectomized mice model was established to evaluate the role of TRIM25 and exosomes in osteoporosis. Exosomes were successfully isolated from BMSCs. BMSCs-derived exosomes upregulated TRIM25 expression, promoting M2 macrophage polarization and osteogenic differentiation. TRIM25 facilitated the ubiquitination and degradation of TREM1. Overexpression of TREM1 reversed the enhanced M2 macrophage polarization and osteogenic differentiation caused by TRIM25 overexpression. TRIM25 enhanced the protective effect of BMSCs-derived exosomes against bone loss in mice. These findings suggested that BMSCs-derived exosomes promoted osteogenic differentiation by regulating M2 macrophage polarization through TRIM25-mediated ubiquitination and degradation of TREM1. This mechanism might provide a novel approach for treating osteoporosis.

**Supplementary Information:**

The online version contains supplementary material available at 10.1186/s10020-024-00904-w.

## Introduction

Osteoporosis, a bone disorder closely linked to aging, is characterized by reduced bone mass, deterioration of bone tissue microarchitecture, decreased bone strength, and an increased risk of fracture (Yu and Wang 2022; Coughlan and Dockery 2014). During adulthood, bone mass is typically maintained at the peak level achieved during adolescence. However, an abnormal bone remodeling cycle can disrupt the balance between bone-forming osteoblasts and bone-resorbing osteoclasts. This disruption leads to a series of physiological and pathological changes, including estrogen deficiency, high glucocorticoid levels, and alterations in serum calcium levels, which collectively result in net bone loss (Coughlan and Dockery 2014). one marrow mesenchymal stem cells (BMSCs) have a robust self-renewal capacity and the ability to differentiate into multiple lineages. They play a critical role as precursors to osteoblastic lineage cells, contributing to the maintenance of the balance between bone formation and bone resorption during bone remodeling (van Gastel and Carmeliet 2021; Zhou et al. 2022). Additionally, BMSCs may improve conditions such as obesity and osteoporosis by balancing osteogenic and adipose differentiation (Guo et al. 2021; Matsushita and Dzau 2017). Risk factors for osteoporosis include aging, early ovariectomy, long-term inactivity, prolonged corticosteroid use, and insufficient calcium intake during youth. Women, especially those over 50 years of age, are significantly more susceptible to developing osteoporosis compared to men. The primary strategies for treating osteoporosis encompass both pharmacological and non-pharmacological interventions, including dietary modifications, exercise, and long-term hormone replacement therapy. However, treatment is often initiated after significant bone loss has occurred, limiting its effectiveness. Given these limitations, there is a need for novel treatments for osteoporosis that warrant further investigation and exploration.

Exosomes (Exos) are extracellular vesicles ranging in size from 40 to 150 nm, containing a variety of components such as nucleic acids, proteins, lipids, amino acids, and metabolites that can reflect their cellular origin (Kalluri and Lebleu 2020). The physiological roles of exosome production are still largely unknown and require further investigation. As research advances, Exos derived from various cell types have shown significant therapeutic potential for treating different diseases (Lu et al. 2020; Li et al. 2021; Zhang et al. 2019). Specifically, the role of Exos derived from BMSCs in bone-related disorders has garnered attention. For example, BMSC-derived exosomal microRNA (miR)-29a has been shown to promote angiogenesis and osteogenesis in osteoporosis (Lu et al. 2020). Similarly, BMSC-derived Exos have been found to protect against cartilage damage and alleviate knee osteoarthritis pain in a rat model (He et al. 2020). Despite these promising findings, the specific mechanisms by which Exos influence osteoporosis and related conditions warrant further exploration.

Macrophages are a heterogeneous population of bone marrow-derived cells within the innate immune system, playing key roles in various physiological and pathological processes in the body (Genin et al. 2015). These cells exhibit two main polarization states: classically activated type 1 (M1) and alternatively activated type 2 (M2). M1 macrophages are characterized by the production of pro-inflammatory cytokines, while M2 macrophages primarily produce anti-inflammatory cytokines. The polarization state of macrophages is reversible, which helps maintain homeostasis. A strong link has been established between macrophage dysfunction and macrophage-induced inflammation, which can contribute to aging and the onset of osteoporosis (Linehan and Fitzgerald 2015). Moreover, macrophage polarization plays a role in promoting osteogenic differentiation and increasing osteogenesis and bone mineralization, suggesting that interventions aimed at reducing the M1/M2 macrophage ratio may be a potential treatment strategy for osteoporosis (Horwood 2016). However, the effects of BMSC-derived Exos on macrophage polarization in osteoporosis have not been thoroughly investigated, representing a promising area for future research.

Ubiquitination is a critical post-translational modification that influences the cellular localization and fate of numerous proteins, thereby impacting a wide range of physiological processes. Dysregulation of ubiquitination can lead to the progression of various diseases, including cancer (Zhu et al. 2022). Tripartite motif (TRIM) proteins are a versatile family of ubiquitin E3 ligases involved in a multitude of cellular and physiological events (van Gent et al. 2018). TRIM25 is a member of the TRIM family and has been implicated in the development of various diseases (Heikel et al. 2016). However, the role of TRIM25 in osteoporosis has not been fully elucidated.

In this study, we aimed to investigate the role of BMSC-derived Exos in osteogenic differentiation and osteoporosis, along with the underlying mechanism. This research is envisioned to provide a novel perspective for treating osteoporosis.

## Methods and materials

### Antibodies

The antibodies used in this study were listed as follows: CD81 (Abcam, Cambridge, MA, USA, ab109201, 1/5000), TSG101 (Abcam, ab125011, 1/5000), CD9 (Abcam, ab236630, 1/1000), TRIM25 (Abcam, ab167154, 1/10000), glyceraldehyde-3-phosphate dehydrogenase (GAPDH; Abcam, ab9485, 1/2500), Runt-related transcription factor 2 (Runx2; Abcam, ab236639, 1/1000), osteocalcin (OCN; Abcam, ab133612, 1/5000), Janus kinase (JAK)1 (Abcam, ab133666, 1/5000), signal transducer and activator of transcription (STAT)6 (Abcam, ab32108, 1/5000), phospho(p)-JAK1 (Abcam, ab138005, 1/5000), p-STAT6 (Abcam, ab263947, 1/1000), p65 (Abcam, ab32536, 1/5000), inhibitor of kappa B alpha (IκBα; Abcam, ab32518, 1/5000), p-p65 (Abcam, ab76302, 1/1000), p-IκBα (Abcam, ab92700, 1/1000), triggering receptor expressed on myeloid cell (TREM)1 (Abcam, ab200729, 1/5), Toll-like receptor (TLR)4 (Abcam, ab13556, 1/1000), ubiquitination (PTM Biotechnology Co., LTD, Hangzhou, China, PTM-1106RM, 1/1000), goat-anti-rabbit secondary antibody (Abcam, ab6721, 1/10000).

### Cell culture

Human BMSCs obtained from Ji’ning Shiye Biotechnology Co. LTD (Shanghai, China) were cultured in Dulbecco’s modified Eagle’s medium (DMEM)/high glucose (HyClone, China) supplemented with 10% fetal bovine serum (FBS, Thermo Fisher Scientific, Waltham, MA, USA), 100 U/mL penicillin and 100 mg/mL streptomycin at 37 °C and 5% CO_2_.

Human osteoblasts were purchased from Procell Life Technology Co., Ltd. (Wuhan, China). The specific protocol for culturing human osteoblasts can be found in the study by (Yang et al. 2022). Human bone marrow-derived macrophages (BMDMs) were also purchased from Procell Life Technology Co., Ltd. (Wuhan, China). They were cultured in a specific culture medium designed for BMDMs, also provided by Procell. Human embryonic kidney (HEK)-293T cells were obtained from Procell Life Technology Co., Ltd. (Wuhan, China). These cells were cultured in Minimum Essential Medium (MEM) supplemented with 10% fetal bovine serum (FBS), 100 U/mL penicillin, and 100 mg/mL streptomycin. All cells were maintained in an incubator set at 37 °C with 5% CO_2_. The culture medium was replaced every three days to ensure optimal growth conditions.

To inhibit the release of Exos from BMDMs, the exosome inhibitor GW4869 (10 µM; Sigma-Aldrich, St. Louis, MO, USA) was added to the culture medium of BMDMs. The cells were then cultured for 48 h at 37 °C with 5% CO_2_, following the protocol described by (Yang et al. 2019). To explore the effects of BMSC-derived Exos on macrophage polarization and osteogenic differentiation, co-culture experiments were performed using a transwell system. BMDMs were co-cultured with BMSCs in a transwell system for 48 h at 37 °C with 5% CO_2_. The transwell system allows for the exchange of soluble factors between the two cell types without direct cell-to-cell contact. BMDMs were co-cultured with human osteoblasts in a transwell system for 48 h at 37 °C with 5% CO_2_. This setup is used to assess the impact of macrophage polarization on osteogenic differentiation.

### BMSCs characterization

To determine the multipotential differentiation capacity of BMSCs, BMSCs were seeded in 12-well plates at a density of 1.5 × 10^4^ cells/cm^2^ in DMEM/high glucose medium. Once the cells reached 80% confluence, the medium was changed to osteogenic differentiation medium. This medium contains: 1 nM dexamethasone, 50 μM L-ascorbic acid-2-phosphate, 20 mM β-glycerophosphate. The osteogenic differentiation medium was purchased from Cyagen Biosciences (Guangzhou, China). After the induction period, the osteogenic differentiation potential was analyzed by Alkaline phosphatase (ALP) staining and Alizarin Red S (ARS) staining. Following the research protocol of Li et al. (Li et al. 2023), BMSCs were cultured in adipogenic differentiation medium. The adipogenic differentiation potential was analyzed by Oil Red O staining to detect lipid droplets.

For detecting stem cell surface markers, BMSCs were were blocked with 3% bovine serum albumin (BSA, Beyotime Biotechnology Co., Ltd, Shanghai, China) for 30 min and stained with antibodies against Sca-1 (ab124688, Abcam), CD44 (BD, USA, 550989), CD-34 (BD, 560710), and CD-45 (BD, 560975) at 4 ℃ for 30 min. Finally, a flow cytometer (Accuri C6, BD Biosciences, San Jose, CA) was applied for the examination of labeled cells.

### ALP staining

The ALP staining assay was performed using a commercial ALP staining kit (Solarbio Biotechnology Co. LTD, Beijing, China) according to the instructions. BMSCs or osteoblasts were washed with PBS and fixed with ALP fixative for 3 min. After that, the cells were incubated with ALP staining solution for 20 min without light. After washing the cells with PBS, osteogenic differentiation of BMSCs was imaged by microscopy, and the osteogenic differentiation capability of osteoblasts was directly photographed.

### ARS staining

ARS staining was performed using a Mineralized nodules Staining kit (Beyotime). BMSCs or osteoblasts were fixed by 4% paraformaldehyde (PFA) for 20 min and then washed by PBS thrice. Next, ARS staining solution was added to the cells to make the cells completely covered to stain the cells at room temperature for 30 min. Finally, the staining results of BMSCs or osteoblasts were analyzed under the microscope or directly photographed, respectively.

### ALP activity detection

Firstly, the osteoblasts were rinsed with PBS thrice, lysed in Radio Immunoprecipitation Assay (RIPA) solution (Beyotime), and finally centrifuged at 12,000 *g* for 10 min at 4 °C to remove cellular debris at 4 °C. Next, ALP activity in the samples was measured by a commercial ALP Assay kit (Beyotime) according to the manufacturer’s protocol. Finally, the staining outcome was analyzed under the microscope.

### Oil red O staining

In brief, 0.05 *g* of oil red O powder (Sigma) was added to 99% propanediol (Sigma) to prepare oil red O dyes. After removing the culture medium, BMSCs were washed with 1 mL of PBS twice. Next, the cells were fixed with 4% PFA in PBS for 15 min at room temperature. After removing the solution, the cells were washed with ddH2O three times, and 0.5% oil red O was added to incubate the cells for 1 h at room temperature. After incubation, the cells were washed with 70% ethanol (Beyotime) three times and analyzed under a microscope.

### Extraction and identification of Exos from human BMSCs

Exos were isolated using sequential ultracentrifugation as described previously (Yue et al. 2022). Initially, BMSC culture medium underwent centrifugation steps at 500 g for 10 min, 2000 g for 10 min, and 10,000 g for 30 min to eliminate cell debris and large organelles. Exos were subsequently collected from the supernatants through ultracentrifugation at 100,000 g for 70 min using a 50.2Ti rotor in a Beckman Coulter ultracentrifuge (Optima XPN-100). Pellets containing Exos were washed in 0.9% NaCl and subjected to another round of centrifugation at 100,000 g for 70 min to further eliminate medium protein contaminants. The exosome pellets were then resuspended in 0.9% NaCl and stored at − 80 °C for subsequent analysis. Characterization of Exos was performed using transmission electron microscopy (TEM), nanoparticle tracking analysis (NTA), and Western blot.

For TEM analysis, Exos were fixed with 2% PFA for 30 min after dilution with PBS, following a method previously described (Yang et al. 2019). The fixed Exos were suspended in PBS and fixed again with 2% PFA for an additional 30 min at room temperature. Subsequently, an 8 μL aliquot of the exosome suspension was loaded onto an EM grid that had been pretreated with ultraviolet light and allowed to incubate for 10 min. The Exos on the grid were then stained twice with 1% uranyl acetate (UA) solution, with each staining session lasting 6 min. Excess UA solution was removed by filter paper, and the grid was allowed to air dry. Finally, the Exos were visualized under a TEM (Hitachi Corp., Tokyo, Japan).

NTA was performed to detect the size of Exos. A small amount of the thawed Exos was added to 1 mL PBS. Their particle sizes were examined by NTA using a NanoSight NS500 device (Malvern Pananalytical, USA) in accordance with a previous study (Jiang et al. 2022).

The presence of exosomal characteristic surface marker proteins (CD81, CD9, and TSG101) were detected by Western blot.

### Cell transfection and treatment

TRIM25 short hairpin RNA (sh-TRIM25), shRNA negative controls (sh-NC), pcDNA-TRIM25 overexpression vector, pcDNA-TREM1 overexpression vector, and empty pcDNA vector used in the present study were obtained from Santa Cruz Biotechnology (CA, USA). The BMDMs (1 × 10^6^ cells/well) were inoculated in six-well plates. Transfection was performed using Lipofectamine 3000 (Thermo Fisher) according to the manufacturer’s instructions after the cell confluence reached about 80%. The cells were transfected for 48 h. Finally, the expression of TRIM25 and TREM1 was analyzed by real-time quantitative polymerase chain reaction (RT-qPCR). After transfection, BMSCs-derived Exos (2 μg of Exos per 1 × 10^5^ recipient cells) were added to the culture medium for 12 h in line with a previous study(Lu et al. 2020).

### Animal studies

A total of 30 adult female BALB/c (~ 20 g B.W; 9 weeks old) mice purchased from Dossy Experimental Animals Co., LTD (Chengdu, China) were housed in cages with 24 ℃, a 12 h alternating light/dark cycle and free access to water and food.

After one-week adaptive feeding, the mice were randomly divided into five groups (n = 5 per group): sham, ovariectomized (OVX), OVX + Exo, OVX + Exo + lentivirus negative control (LV-NC), and OVX + Exo + TRIM25 overexpression lentivirus (LV)-TRIM25. LV-NC and LV-TRIM25 (Santa Cruz Biotechnology) were injected into tail vein of mice after one week of model establishment. Next, the BMSC-Exos suspension (20 mg of Exos/mouse) was injected twice per week by intra-femoral injection. The establishment of OVX model was performed according the published procedure (Yang et al. 2019). Briefly, the mice were anaesthetized with pentobarbital sodium (30 mg/kg, Sigma), and then two bilateral incision (10 mm diameter) was made on lateral lumbar skin. The bilateral ovaries of the mice were cautiously removed by exposing the muscles and retroperitoneum by blunt dissection. The animals in the sham group received the same procedure without the removal of bilateral ovaries. The tissue was then repositioned and sutured. The mice were injected with 40,000 IU/mL penicillin at 1 mL/kg for 3 days. 2 months after OVX model establishment, the femur was obtained to confirm the occurrence of osteoporosis using micro-computed tomography (micro-CT) analysis.

### micro-CT

Bone microarchitecture analysis of trabecular bone was performed using a micro-CT system (mCT-80, Scanco Medical, Brüttisellen, Switzerland), as previously described (Sun et al. 2022). The imaging parameters were set as follows: voxel size of 9 μm, voltage of 55 kV, and current of 70 mA. Following the scan, the region of interest (ROI) was selected between 0.3 to 0.6 mm from the highest point on the distal femur growth plate.

Subsequently, 3D images were analyzed to assess various bone parameters, including bone volume/tissue volume (BV/TV), trabecular thickness (Tb.Th), trabecular number (Tb.N), and trabecular separation (Tb.Sp).

### Isolation and quantification of RNAs

Firstly, the commercial FastPure Cell/Tissue total RNA isolation kit V2 (Vazyme Biotechnology Co., LTD, Nanjing, China) was used to extract total RNA from cells. Then, the extracted RNA was reverse transcribed into cDNA using the Hifair^®^ V one-step RT-gDNA digestion SuperMix for qPCR kit (Yeason Biotechnology, Shanghai, China), and the qPCR amplification experiment was performed using the Hieff^®^ qPCR SYBR Green Master Mix kit (Yeason) with the reaction conditions: 95 °C for 5 min, 40 cycles of 95 °C for 10 s, 60 °C for 20 s, and 72 °C for 20 s and a melt curve stage. The gene expression was calculated by the 2^−ΔΔCT^ method. Primers used in this study were synthesized by Geenseed Biotechnology Co., LTD (Guangzhou, China) and listed as follows: inducible nitric oxide synthase (iNOS), forward, 5′—CCAGCTAGCCAAAGTCACCAT—3′ and reverse, 5′—GTCTCGGAGCCATACAGGATT-3′; tumor necrosis factor-α (TNF-α), forward, 5′—GAGCAAGCCCTGGTATG—3′ and reverse, 5′—CGGGCCGATGAGATGATCTCTCG—3′; interleukin (IL)—6, forward, 5′—TGCAATAACCCCTGACC—3′ and reverse, 5′—ATTTGCCGAAGCCCG—3′; mannose receptor C-type 1 (MRC1) (CD206), forward, 5′—TGATACCTGCGACAGTAAACGA—3′ and reverse, 5′—CTTGCAGTATGTCTCCGCTTC-3′; IL-10, forward, 5′-GCCAAGCCTTGTCTGAGATGATCC-3′ and reverse, 5′—AATCGATGACAGCGCCGTAGC—3′; arginase 1 (Arg1), forward, 5′—GTGGAAACTTGCATGGACAAC-3′ and reverse, 5′—AATCCTGGCACATCGGGAATC-3′; tripartite motif (TRIM) 16, forward, 5′—GTCCTGTCTAACCTGCATGGT-3′ and reverse, 5′—GGCAGTATCGCCAGTTGTG—3′; TRIM21, forward, 5′-TCAGCAGCACGCTTGACAAT-3′ and reverse, 5′—GGCCACACTCGATGCTCAC—3′; TRIM25, forward, 5′—AATCGGCTGCGGGAATTTTTC—3′ and reverse, 5′-TCTCACATCATCCAGTGCTCT—3′; TRIM38, forward, 5′—GAGCCTGATGACGAACCCAG-3′ and reverse, 5′—TCTTGATCCGTCTCTTTGAGGG—3′; TRIM62, forward, 5′—CGAGCAGCATCAGGTCACC—3′ and reverse, 5′—CCAGTTGTCGCTTGAGCAG-3′; triggering receptor expressed on myeloid cell 1 (TREM1), forward, 5′—GAACTCCGAGCTGCAACTAAA-3′ and reverse, 5′-TCTAGCGTGTAGTCACATTTCAC—3′; glyceraldehyde-3-phosphate dehydrogenase (GAPDH), 5′—GACTCATGACCACAGTCCATGC—3′ and reverse, 5′—AGAGGCAGGGATGATGTTCTG—3′.

### Western blot

Cells were lysed with RIPA lysis buffer for 30 min until complete lysis occurred. The lysate was then homogenized at 4 °C for 30 min and centrifuged at 12,000 rpm for 20 min at 4 °C. The resulting supernatant was carefully collected and stored at − 80 °C until use. Protein concentration was determined using the Bradford assay (Sigma). Subsequently, 50 μg of protein was separated by 10% SDS-PAGE (Thermo Fisher). Prior to use, the PVDF membrane was briefly soaked in pure methanol (Beyotime) for 5 s and then used for protein transfer. To minimize non-specific binding, the membrane was blocked with 5% BSA at 37 °C for 1 h. Following blocking, the membrane was incubated with primary antibodies (Abcam) overnight at 4 °C. After washing the membrane three times (10 min each) with Tris-buffered saline Tween (TBST, Beyotime), it was incubated with secondary antibodies (Abcam) at room temperature for 2 h. Finally, protein signals were detected using an enhanced chemiluminescence solution (Yeason).

### Hematoxylin–eosin (H and E) staining

Femur tissues isolated from mice were first fixed in 4% PFA for 24 h, followed by decalcification in 15% ethylenediaminetetraacetic acid (EDTA, Yeason) for 21 days. After decalcification, the tissues were embedded in paraffin (Aladdin Biochemical Technology Co., LTD, Shanghai, China). Paraffin-embedded tissues were then sectioned serially into 4 μm thick sections. To prepare for staining, the sections were dewaxed in xylene (Sigma) and rehydrated through a series of ethanol gradients (95%, 90%, 80%, and 70%). For staining, the tissue sections were immersed in hematoxylin (Sigma) for 5 min, rinsed in running water, differentiated in 1% ethanol for 30 s, blueing in 0.6% ammonia water until the desired hue was achieved, and washed again in running water. Subsequently, the sections were stained with eosin (Sigma) for 3 min. Finally, after dehydration, the sections were mounted with neutral balsam mounting medium (Sangon Biotechnology, Shanghai, China), and images were acquired using a biopathology microscope (BX45-DP72, Olympus, Japan).

### Co-immunoprecipitation (Co-IP) assay

The interaction between TRIM25 and TREM1 was assessed using the Co-IP assay following methods described previously (Luo et al. 2023). Cells were lysed on ice in RIPA buffer supplemented with a protease inhibitor (aprotinin, Yeason) for 20 min, followed by centrifugation at 12,000 *g* for 10 min at 4 °C. The resulting supernatant was collected, and 20 μL was reserved as the input sample, to which 2 × SDS sample buffer was added and boiled for 5 min. The remaining cell lysates were incubated overnight at 4 °C with specific antibodies, followed by the addition of protein A/G beads (Santa) and further incubation for 2 h. Subsequently, the beads were washed five times with cell lysis buffer, and the bound proteins were eluted using 2 × SDS sample buffer and boiled at 100 °C for 5 min. The eluates were then subjected to Western blot analysis.

### Ubiquitination assay

IP assay combined with Western blot were used to access the ubiquitination level of TREM1 in HEK-293T cells. Briefly, the HEK-293T cell lysates were obtained and immunoprecipitated with anti-TREM1 antibody and protein A/G agarose (Santa), followed by Western blot analysis with the primary antibody against ubiquitin (PTM Biotechnology Co., LTD, Hangzhou, China).

### Protein stability assessment

Protein stability assessment was performed to verify the protein stability of TREM1 after TRIM25 overexpression in HEK-293T cells. The cells were treated with cycloheximide (CHX, 100 μg/mL, Abcam), and the protein level of TREM1 at different time points (0, 2, 4, and 8 h) was detected.

### Statistical analysis

SPSS 21.0 software was used to analyze data. Data are expressed as mean ± standard deviation (SD). Student’s t-test was used for comparison between the two groups. One-way analysis of variance (ANOVA) was used for comparison among groups. Statistical analyses were performed using GraphPad Prism software (v8.0.1, GraphPad Software Inc., San Diego, CA, USA). *p* < 0.05 indicates that the difference is statistically significant.

## Results

### Identification of BMSCs and BMSCs-derived Exos

**Fig. 1:**
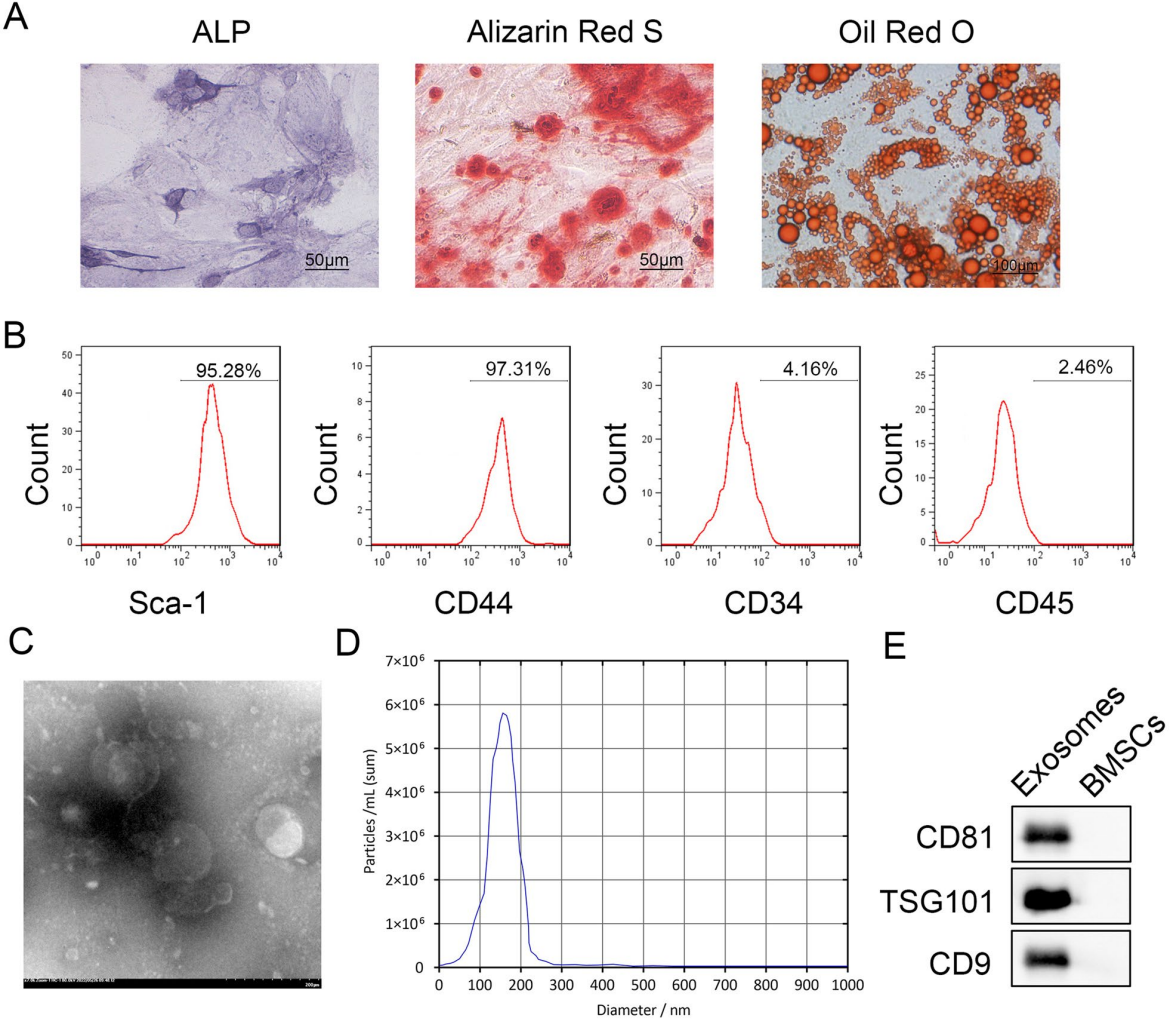
Identification of BMSCs and BMSCs-derived Exos. **A**, Alkaline phosphatase (scale bar = 50 μm), Alizarin red S (scale bar = 50 μm), and oil red O staining (scale bar = 100 μm) were used to determine the multipotential differentiation capabilities of BMSCs; **B**, Characteristic surface markers of BMSCs evaluated by flow cytometry; **C**, The morphology of exosome (200 μm) was observed under a TEM; **D**, Nanoparticle tracing assay of the BMSCs-derived Exos; **E**, The protein levels of CD81, CD9, and TSG101 were measured by Western blot. *TEM*, transmission electron microscopy, *BMSC*, bone marrow mesenchymal stem cell, *Exo* exosome

In the identification experiment of BMSCs, we observed significant ALP staining, calcium nodules, and lipid droplets (Fig. 1A), suggesting that the cells have the potential for multidirectional differentiation. Moreover, we measured the surface markers of BMSCs, including Sca-1, CD44, CD34, and CD45, and the results showed that the cells expressed Sca-1 and CD44, but barely expressed CD34 and CD45 (Fig. 1B), confirming that BMSCs met the criteria for identifying stem cells. We then isolated Exos from BMSCs and identified them using TEM, NTA, and Western blot. The results indicated that the Exos were manifested as bilayer membrane cup-shaped or circular vesicles (Fig. 1C). Furthermore, the diameter of most Exos ranged between 100 and 200 nm (Fig. 1D). CD81, CD9, and TSG101 are representative surface marker proteins of Exos (Hu et al. 2020). As shown in Fig. 1E, CD81, TSG101, and CD9 were expressed in Exos but not in BMSCs, implying that we successfully isolated Exos from BMSCs.

### BMSCs-derived Exos promoted M2 macrophage polarization

**Fig. 2:**
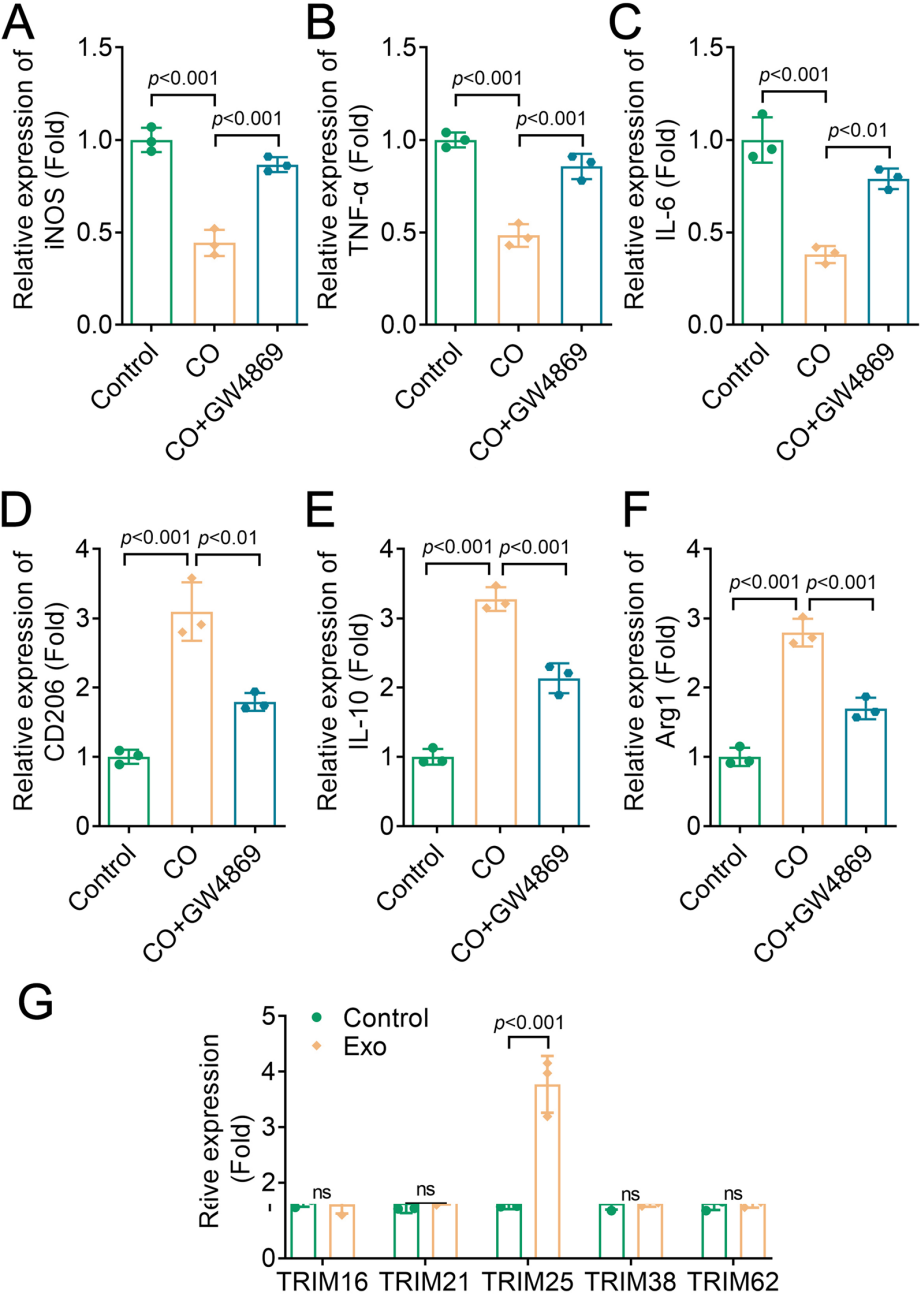
BMSCs-derived Exos promoted M2 macrophage polarization. The expression of **A**, iNOS, **B**, TNF-α, **C**, IL-6, **D**, CD206, **E**, IL-10, **F**, Arg1, and **G**, TRIM family genes were analyzed by RT-qPCR. iNOS, inducible nitric-oxide synthase; *TNF-α* tumor necrosis factor-α, *RT-qPCR* real-time quantitative polymerase chain reaction, *IL* interleukin, *Arg1* arginase1, *TRIM* tripartite motif, *Exo* exosome, *CO* co-culture, *BMSC* bone marrow mesenchymal stem cell

To investigate the effect of BMSC-derived Exos on macrophage polarization, we used GW4869, an inhibitor of exosome release, in a co-culture system of BMSCs and BMDMs. The expression of M1 and M2 surface markers were then assessed. The results indicated that the expression levels of M1 markers (iNOS, TNF-α, and IL-6) were decreased, while those of M2 markers (CD206, IL-10, and Arg1) were increased in the co-culture group. These changes were reversed after GW4869 treatment (Fig. 2A–F). These findings suggested that BMSC-derived Exos promoted M2 macrop hage polarization.

A previous study has shown that BMSC-derived Exos regulate ubiquitination to promote fracture healing in mice (Jiang et al. 2020). Therefore, we explored whether ubiquitination modification occurs in BMSC-derived Exos. We assessed the expression of a series of ubiquitination enzymes in the TRIM family. The data showed that TRIM25 was upregulated in BMDMs treated with Exos compared to the control group, while the expression of TRIM16, TRIM21, TRIM38, and TRIM62 showed no significant differences between the two groups (Fig. 2G). These results suggested that exosomal TRIM25 might promote macrophage M2-type polarization.

### Exosomal TRIM25 promoted osteogenic differentiation by promoting M2 macrophage polarization

**Fig. 3:**
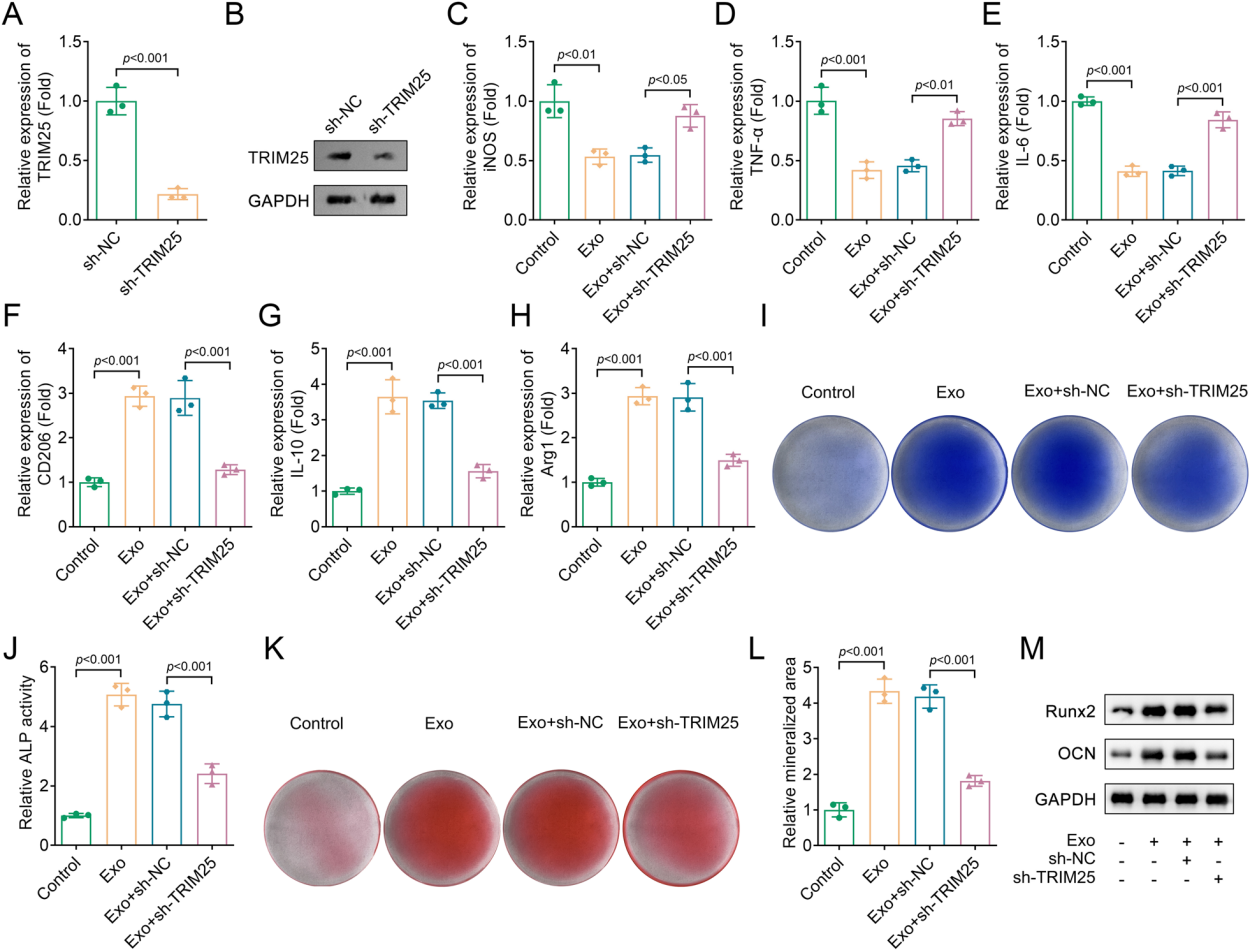
Exosomal TRIM25 promoted osteogenic differentiation by promoting M2 macrophage polarization. The expression of TRIM25 in sh-NC and sh-TRIM25 groups was detected by **A**, RT-qPCR and **B**, Western blot; **C**, iNOS, **D**, TNF-α, **E**, IL-6, **F**, CD206, **G**, IL-10, and **H**, Arg1 expression in each group were analyzed by RT-qPCR; **I**, ALP staining was performed to assess osteogenic differentiation; **J**, ALP activity was measured using a commercial kit; **K**, Alizarin red S staining was performed to detect the mineralized area in each group; **L**, Quantification of mineralized area in each group; **M**, The protein levels of Runx2 and OCN in each group were detected by Western blot. *TRIM* tripartite motif, *RT-qPCR* real-time quantitative polymerase chain reaction; *iNOS* inducible nitric-oxide synthase, *TNF-α* tumor necrosis factor-α, *IL*, interleukin, *Arg1* arginase1, *Runx2* Runt-related transcription factor 2, *OCN* osteocalcin, *ALP* alkaline phosphatase, *shRNA* short hairpin RNA, *Exo* exosome

To investigate the effects of TRIM25 on macrophage polarization, we transfected sh-TRIM25 into BMDMs. The results showed a significant inhibition of TRIM25 expression (Fig. 3A, B). Macrophage polarization was assessed using RT-qPCR. Silencing TRIM25 reversed the downregulated M1 markers (iNOS, TNF-α, and IL-6) and the upregulated M2 markers (CD206, IL-10, and Arg1) in the BMSC-derived Exo group (Fig. 3C–H). This suggested that BMSC-derived Exos promoted M2 macrophage polarization by regulating the expression of TRIM25. Furthermore, exosome-treated BMDMs were co-cultured with osteoblasts to evaluate osteogenic differentiation. We found that the Exo group exhibited higher ALP staining, ALP activity, and mineralized area compared to the control group, while these results were restored after TRIM25 inhibition (Fig. 3I–L). Runx2 is a basic transcription factor that stimulates osteogenesis, and osteocalcin (OCN) is a protein secreted by osteoblasts. The results of Western blot showed that inhibiting TRIM25 reduced the increased protein levels of Runx2 and OCN in the Exo group (Fig. 3M). These results indicated that silencing TRIM25 inhibited the ability of osteogenic differentiation mediated by exosome-treated BMDMs.

### Overexpressing TRIM25 promoted the effects of BMSCs-derived Exos on M2 macrophage polarization and osteogenic differentiation

**Fig. 4:**
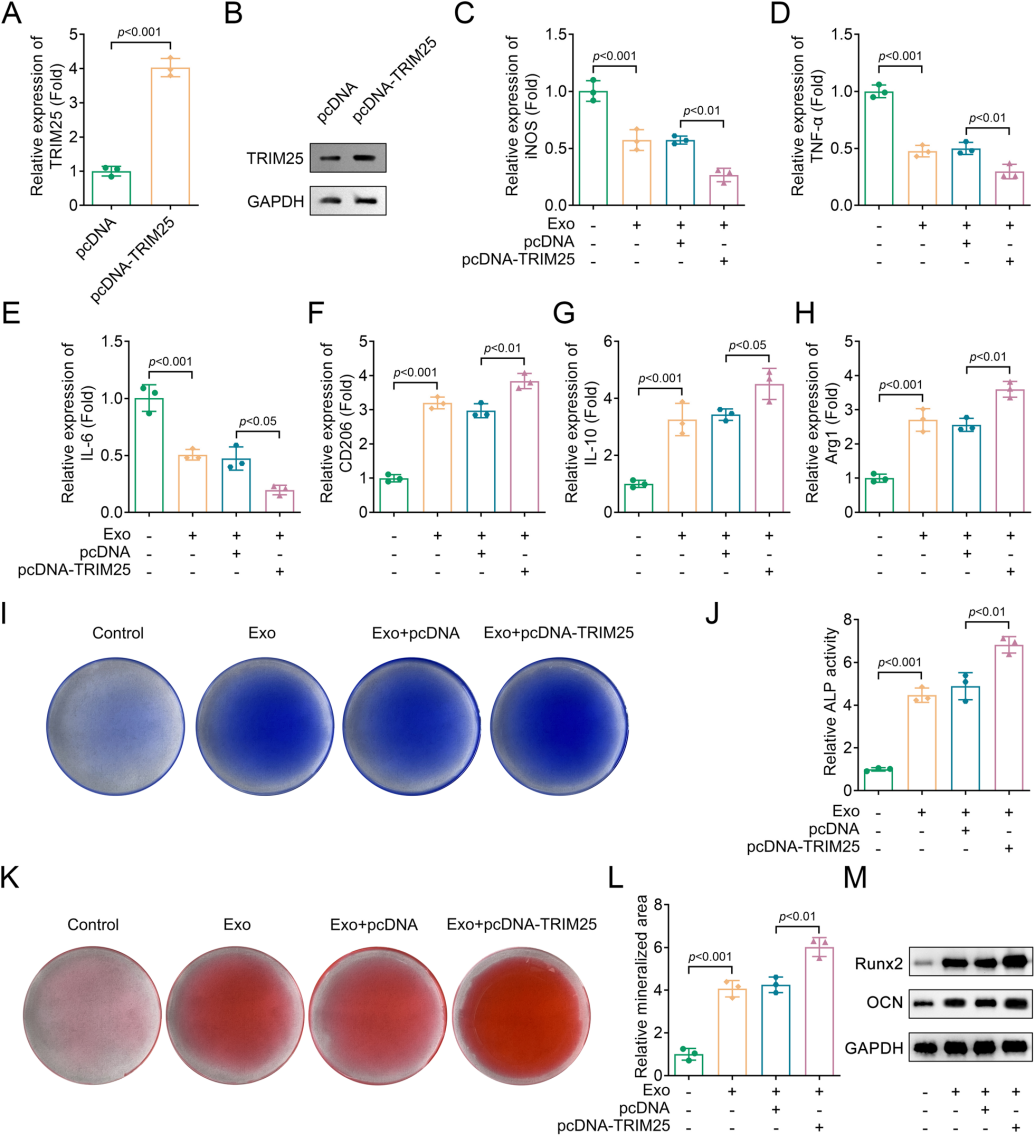
Overexpressing TRIM25 promoted the effects of BMSCs-derived Exos on M2 macrophage polarization and the ability of osteogenic differentiation. The expression of TRIM25 in pcDNA and pcDNA-TRIM25 groups was detected by **A**, RT-qPCR and **B**, Western blot; The expression of **C**, iNOS, **D**, TNF-α, **E**, IL-6, **F**, CD206, **G**, IL-10, and **H**, Arg1 in each group were analyzed by RT-qPCR; **I**, ALP staining was performed to assess osteogenic differentiation; **J**, ALP activity was measured using a commercial kit; **K**, Alizarin red S staining was performed to detect the mineralized area in each group; **L**, Quantification of mineralized area in each group; **M**, The protein levels of Runx2 and OCN in each group were detected by Western blot. BMSC, bone marrow mesenchymal stem cell; *TRIM* tripartite motif, *RT-qPCR* real-time quantitative polymerase chain reaction, *iNOS* inducible nitric-oxide synthase, *TNF-α* tumor necrosis factor-α, *IL*, interleukin; *Arg1* arginase1, *Runx2* Runt-related transcription factor 2, *OCN* osteocalcin, *ALP* alkaline phosphatase, *Exo* exosome

To further investigate the effects of TRIM25 overexpression on macrophage polarization and osteogenic differentiation, we transfected the pcDNA-TRIM25 vector into BMDMs. The results showed a significant upregulation of TRIM25 expression (Fig. 4A, B). Besides, overexpressing TRIM25 downregulated the expression of M1 markers (iNOS, TNF-α, and IL-6) and upregulated the expression of M2 markers (CD206, IL-10, and Arg1) compared to the control group (Fig. 4C–H). These findings suggested that overexpression of TRIM25 promoted M2 macrophage polarization. Additionally, we found that TRIM25 overexpression increased ALP staining, ALP activity, and the mineralized area in comparison with the BMSC-derived exosome (Exo) group in a BMDMs and osteoblasts co-culture study (Fig. 4I–L). The Western blot results showed that overexpressing TRIM25 increased the protein levels of Runx2 and OCN compared to the Exo group (Fig. 4M). These results suggested that overexpressing TRIM25 enhanced the promotive effects of BMSC-derived Exos on M2 macrophage polarization and the ability of osteogenic differentiation.

### TRIM25 interacted with TREM1 to ubiquitinate TREM1 in HEK-293T cells

**Fig. 5:**
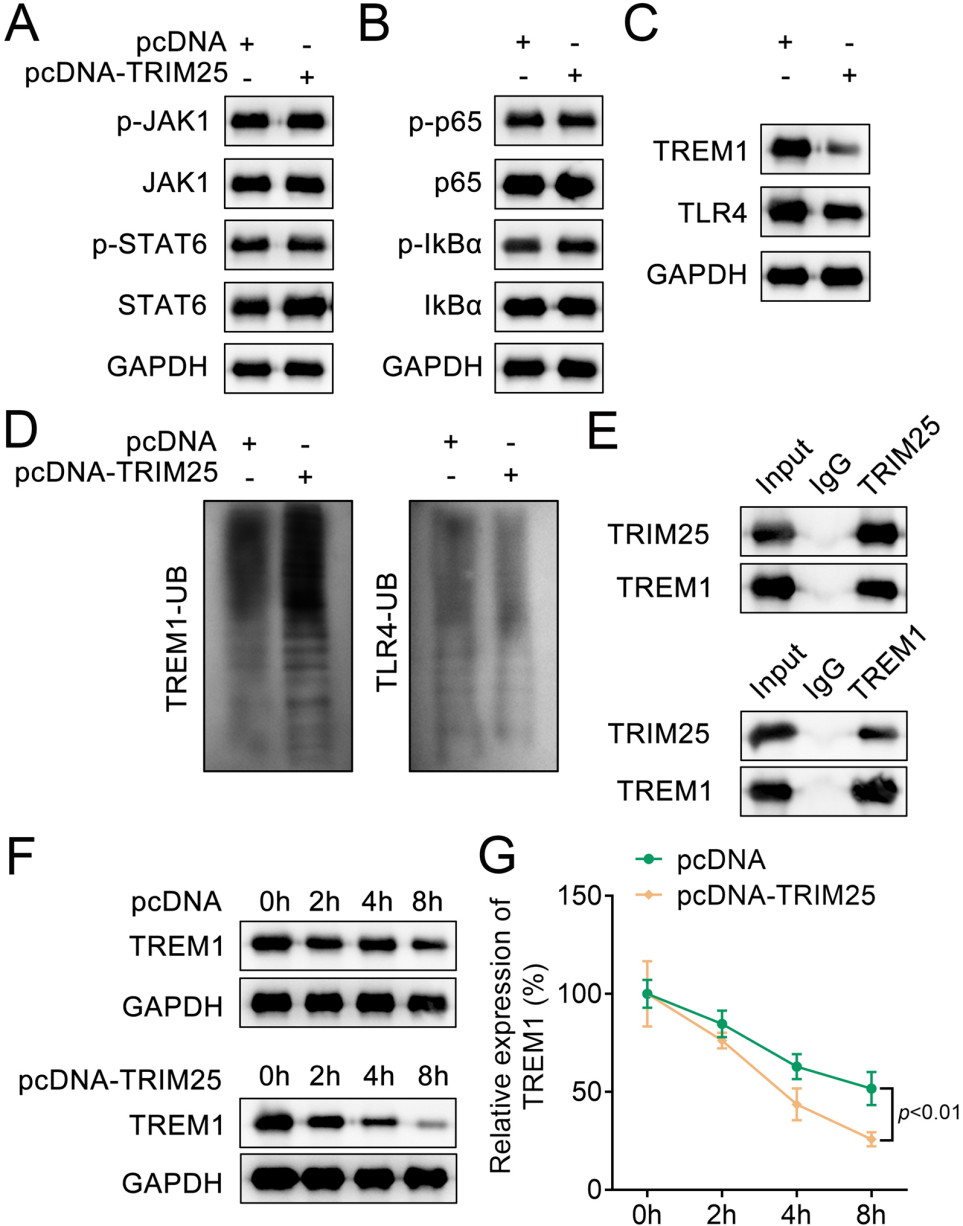
TRIM25 interacted with TREM1 to ubiquitinate TREM1 in HEK-293T cells. **A**, Western blot was performed to detect the protein levels of p-JAK1, JAK1, p-STAT6, and STAT6 after TRIM25 overexpression in HEK-293T cells; **B**, The protein levels of p-p65, p65, p-IκBα, and IκBα in pcDNA and pcDNA-TRIM25 groups were analyzed by Western blot; **C**, The protein levels of TREM1 and TLR4 were analyzed by Western blot; **D**, Western blot was performed to assess the TREM1-UB and TLR4-UB protein levels in HEK-293T cells; **E**, Co-IP assay was conducted to detect the interaction between TRIM25 and TREM1 in HEK-293T cells; **F**, The protein stability assay was used to detect the existing TREM1 protein level at different time points (0, 2, 4, and 8 h) in HEK-293T cells; **G**, Quantification of the existing TREM1 protein level at different time points (0, 2, 4, and 8 h) in HEK-293T cells. *JAK1* Janus kinase-1, *p-JAK1* phosphorylated JAK1, *STAT6* signal transducer and activator of transcription 6, *p-STAT6* phosphorylated STAT6, *HEK* human embryonic kidney, *Co-IP* co-immunoprecipitation, *TRIM* tripartite motif, *IκBα* inhibitor of kappa B alpha, *UB* ubiquitination, *TREM1* triggering receptor expressed on myeloid cells 1, *TLR4* Toll-like receptor 4, *RT-qPCR* real-time quantitative polymerase chain reaction

Previous studies have reported that the JAK1/STAT6, NF-κB, and TREM1/TLR4 pathways play important roles in bone development, physiological, and pathological processes (Du et al. 2021; Chen et al. 2022; Bostanci et al. 2019; Zeng et al. 2022; Sims 2020). Therefore, we examined the protein levels of key factors in these pathways. Western blot results illustrated that the TREM1 and TLR4 protein levels were downregulated, while other proteins had no significant differences after TRIM25 overexpression (Fig. 5A–C). Next, we further detected the ubiquitination levels of TREM1 and TLR4 and found that the TREM1 ubiquitination level was enhanced, while that of TLR4 was not altered in the pcDNA-TRIM25 group compared to the pcDNA group (Fig. 5D). Co-IP results showed that TRIM25 interacted with TREM1 in HEK-293T cells (Fig. 5E). Protein stability assessments revealed that overexpression of TRIM25 resulted in an accelerated degradation of TREM1 protein in HEK-293T cells (Fig. 5F, G). In summary, TRIM25 promoted TREM1 ubiquitination, leading to accelerated TREM1 protein degradation.

### Overexpressing TREM1 reversed the increased M2 macrophage polarization and osteogenic differentiation caused by overexpressing TRIM25

**Fig. 6:**
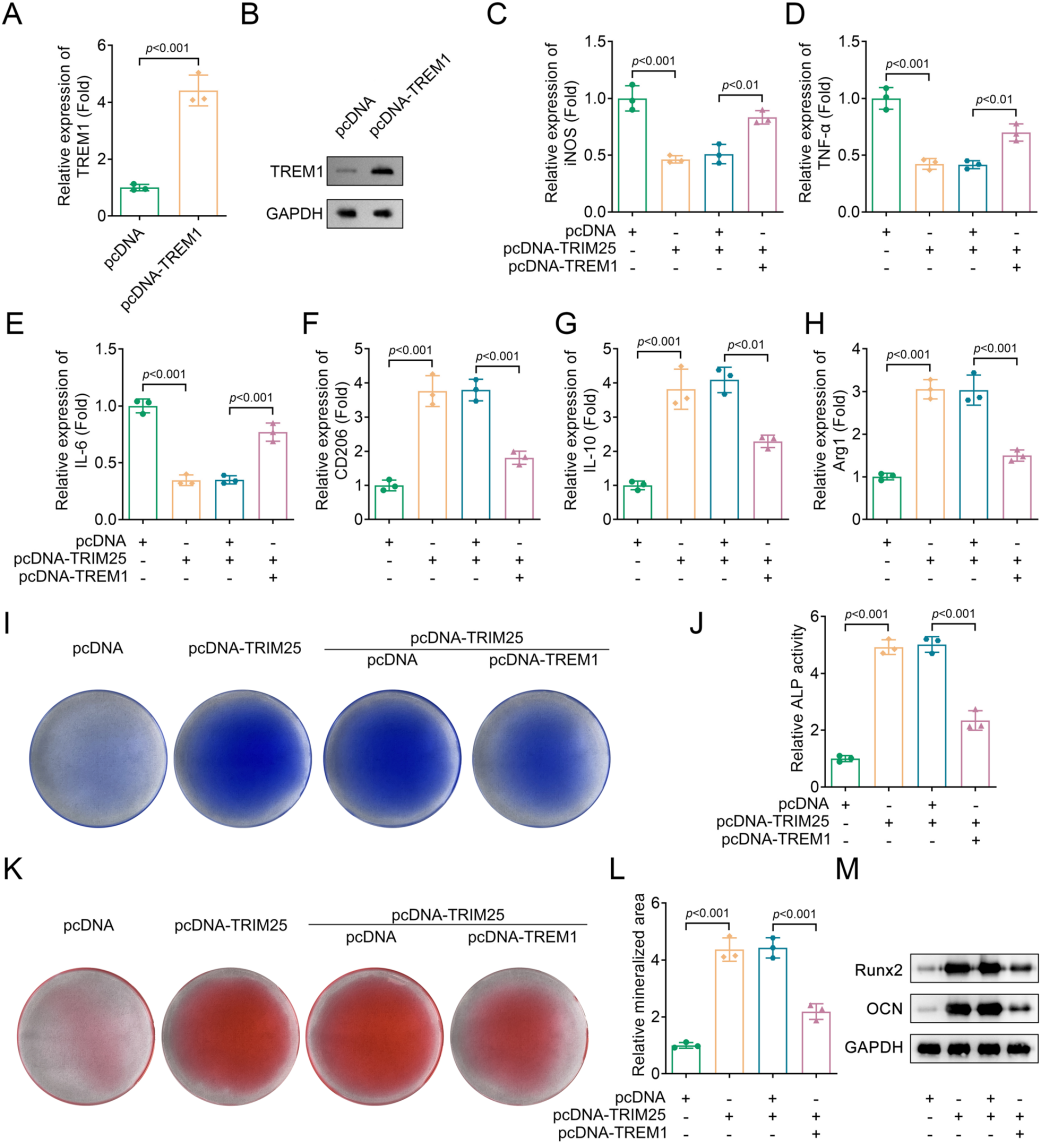
Overexpressing TREM1 reversed the increased M2 macrophage polarization and osteogenic differentiation ability caused by overexpressing TRIM25. The expression of TREM1 in pcDNA and pcDNA-TREM1 groups was detected by **A**, RT-qPCR and **B**, Western blot; The expression of **C**, iNOS, **D**, TNF-α, **E**, IL-6, **F**, CD206, **G**, IL-10, and **H**, Arg1 in each group were analyzed by RT-qPCR; **I**, ALP staining was performed to assess osteogenic differentiation; **J**, ALP activity was measured using a commercial kit; **K**, Alizarin red S staining was performed to detect the mineralized area in each group; **L**, Quantification of mineralized area in each group; **M**, The protein levels of Runx2 and OCN in each group were detected by Western blot. *TRIM* tripartite motif, *RT-qPCR* real-time quantitative polymerase chain reaction, *iNOS* inducible nitric-oxide synthase, *TNF-α* tumor necrosis factor-α, *IL* interleukin, *Arg1* arginase1, *Runx2* Runt-related transcription factor 2, *OCN* osteocalcin, *TREM1* triggering receptor expressed on myeloid cells 1; ALP, alkaline phosphatase

To further verify the effects of TREM1 on macrophage polarization and osteogenic differentiation, we transfected pcDNA and pcDNA-TREM1 vectors into BMDMs. The results showed a significant upregulation of TREM1 mRNA and protein levels (Fig. 6A, B). Overexpressing TREM1 reversed the downregulated M1 markers (iNOS, TNF-α, and IL-6) and the upregulated M2 markers (CD206, IL-10, and Arg1) expression in the pcDNA-TRIM25 group (Fig. 6C–H). These findings suggested that TREM1 plays a role in modulating the effects of TRIM25 on macrophage polarization.In a BMDMs and osteoblasts co-culture study, we found that the pcDNA-TRIM25 group presented higher ALP activity and mineralized area compared to the control group, while these results were restored after TREM1 overexpression (Fig. 6I–L). Moreover, Western blot results indicated that overexpressing TREM1 decreased the increased protein levels of Runx2 and osteocalcin (OCN) induced by TRIM25 overexpression (Fig. 6M). These findings suggested that TREM1 overexpression counteracted the effects of TRIM25 on osteogenic differentiation.

### BMSC-derived exosomal TRIM25 attenuated bone loss in mice caused by OVX

**Fig. 7:**
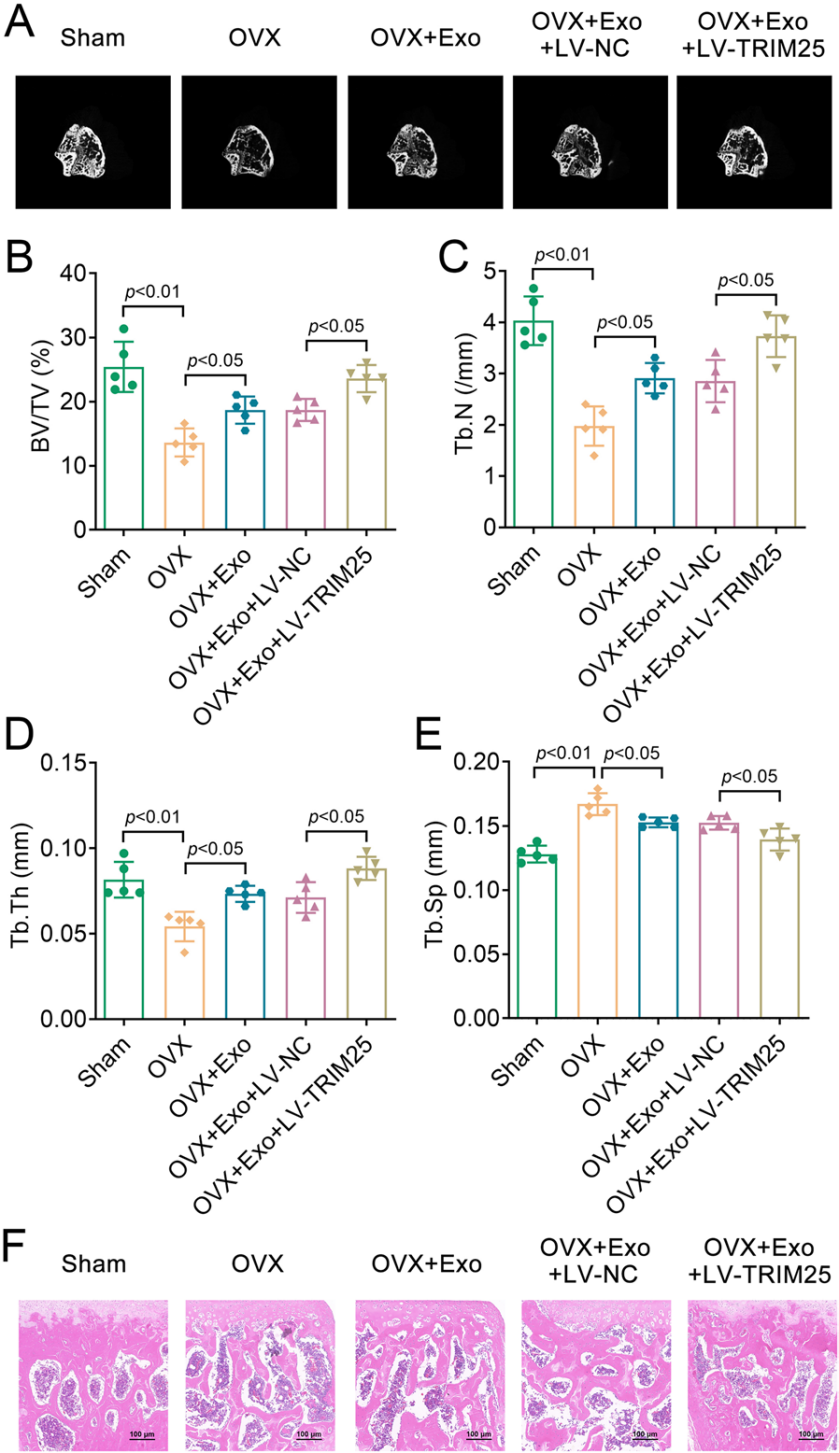
TRIM25 treatment enhanced the effect of BMSCs-derived Exos on resisting bone loss in mice. **A**, Three-dimensional CT image of femur; Quantitative data of **B**, BV/TV, **C**, Tb.N, **D**, Tb.Th, and **E**, Tb.Sp of femurs in mice; **F**, H&E staining was performed to detect the bone mass in each group mice (scale bar = 100 μm). *TRIM25* tripartite motif 25, *BMSC* bone marrow mesenchymal stem cell, *CT* computed tomography, *BV/TV* bone volume/tissue volume, *Tb.N* trabecular number, *Tb.Th* trabecular thickness, *Tb.Sp* trabecular separation, H&E, hematoxylin–eosin; *Exo*, exosome

To investigate the modulatory effect of BMSC-derived Exos on the progression of osteoporosis by regulating TRIM25, we conducted an in vivo study using OVX mice to mimic menopause-induced osteoporosis in women. Exos were injected into mice to explore their effects on osteoporosis progression. The results showed that the trabecular bone was significantly decreased in the OVX group compared to the Sham group, while the OVX + Exo group showed increased trabecular bone compared to the OVX group. Furthermore, the LV-TRIM25 group showed increased trabecular bone in comparison with the LV-NC (negative control) group (Fig. 7A). Quantitative analysis of microcomputed tomography data revealed that, compared with the sham group, the OVX group reduced BV/TV, Tb.N, and Tb.Th, as well as increased Tb.Sp; these results were reversed by Exos treatment. Compared with the LV-NC group, the LV-TRIM25 group showed upregulated BV/TV, Tb.N, and Tb.Th, as well as downregulated Tb.Sp (Fig. 7B–E). H and E staining results indicated that OVX induced severe bone mass loss in mice, which was significantly rescued after Exo treatment. In addition, compared with the LV-NC group, the LV-TRIM25 group showed improved bone mass (Fig. 7F). Collectively, these data revealed that TRIM25 treatment enhanced the effect of BMSC-derived Exos on resisting bone loss in mice.

## Discussion

Recent strategies for treating osteoporosis have focused on influencing the activity of bone stem cells and osteoblasts, which have been shown to be effective in promoting bone formation (Marie and Kassem 2011). Exos possess inherent endo- and heterogeneity, granting them distinct advantages including low immunogenicity, excellent stability, and convenient storage. Therefore, Exos are considered to be promising tools in the field of disease diagnosis and treatment (Zhang et al. 2020; Zhou et al. 2022). Furthermore, BMSC-derived Exos show great potential in the treatment of bone metabolic diseases (Zhou et al. 2022). In this study, we successfully isolated Exos from BMSCs and found that BMSC-derived Exos promoted M2 polarization of macrophages. These findings are consistent with previous research showing that systemically infused BMSCs enhance wound healing by promoting macrophages toward M2 polarization (He et al. 2019). Many studies have also found that ubiquitination modification promotes M2 polarization of macrophages (Pan et al. 2022; Liang et al. 2019). Thus, we detected the expression of a series of ubiquitin enzymes and found that TRIM25 was upregulated in exosome-internalized BMDMs. Moreover, TRIM25 knockdown inhibited M2 macrophage polarization and the ability of osteogenic differentiation. Similarly, a previous review indicates that polarization of macrophages is connected with bone regeneration (Zhou et al. 2022). A similar study demonstrated that TRIM21 expression is significantly increased in bone tissue of osteoporosis patients (Liu et al. 2023). Additionally, TRIM38 has been found to be involved in osteoclast and osteoblast differentiation (Kim et al. 2018).

TREM proteins are a family of cell surface receptors involved in a variety of cellular processes, including inflammation, bone homeostasis, and neurodevelopment. TREM1, the first member to be discovered, amplifies inflammation (Klesney-Tait et al. 2006). The role of TREM1 in liver-related diseases and periodontitis has been extensively studied (Bostanci et al. 2019; Sun et al. 2020). However, its role in bone-related diseases has been poorly studied. In the present study, we found that TRIM25 interacted with TREM1 to ubiquitinate it in HEK-293T cells. This is the first discovery of the ubiquitination modification of TREM1 by TRIM25. In the functional validation experiments of TREM1, overexpressing TREM1 reversed the increased M2 macrophage polarization and osteogenic differentiation ability caused by overexpressing TRIM25. The relationship between TRIM25 and TREM1 in BMDMs has not been explored before. A similar study indicates that after TREM1 gene knockout, macrophage infiltration in BMDMs is limited, and the levels of M1 macrophage-related genes are decreased (Wu et al. 2022). In addition, a growing number of studies have found that TREM1 influences the progression of various diseases by regulating macrophage polarization (Wu et al. 2022; Zhong et al. 2023; Liu et al. 2023). However, the effect of TREM1 on osteogenic differentiation has not been well established. Taken together, our findings suggested that BMSC-derived Exos promoted M2 macrophage polarization and osteogenic differentiation via TRIM25-mediated ubiquitination of TREM1. This mechanism provided a new insight into the role of TREM1 in bone metabolism and opens potential avenues for therapeutic interventions in bone-related diseases.

OVX is a common animal model used for simulating postmenopausal osteoporosis (Geng et al. 2019). Effects of BMSCs-derived Exos and TRIM25 on osteoporosis were further investigated in vivo in an OVX model study. In this present study, BMSCs-derived Exos prevented osteoporotic bone loss in mice, and this effect was enhanced after TRIM25 overexpression. The results were consistent with that in in vitro results. Similar studies have also found that Exos from different sources have the effect of alleviating osteoporosis in OVX in vitro models (Qi et al. 2016; Sadat-Ali et al. 2021).

However, limitations are existed in this study. For example, the ubiquitination sites in TREM1 remain unknown. In addition, we have not yet evaluated downstream target of TRIM25 in vivo study. Moreover, to definitively demonstrate a direct BMDM effect on osteoblasts, further in vivo studies are needed. We will further resolve these limitations in our future work.

In conclusion, the results of this study showed that BMSCs-derived Exos promoted M2 macrophage polarization via TRIM25-mediated ubiquitination of TREM1. Thus, osteogenic differentiation was facilitated and osteoporosis was alleviated. This finding provides a broader understanding of the pathogenesis of osteoporosis and suggests novel therapeutic strategies for its treatment. Further research is warranted to elucidate the specific ubiquitination sites in TREM1, evaluate the downstream targets of TRIM25, and confirm the direct effects of BMDMs on osteoblasts in vivo.

## Supplementary Information

Supplementary material 1. Mechanism idea diagram. A, Experimental flow chart; B, Experimental mechanism chart.

## Data Availability

The datasets used and/or analyzed during the current study are available from the corresponding author on reasonable request.
