# Supplementary figures and images for: BMSC-derived exosomes promote osteoporosis alleviation via M2 macrophage polarization
Source: Mol Med. 2024 Nov 19;30:220. doi: 10.1186/s10020-024-00904-w (PMC11577737; doi:10.1186/s10020-024-00904-w)

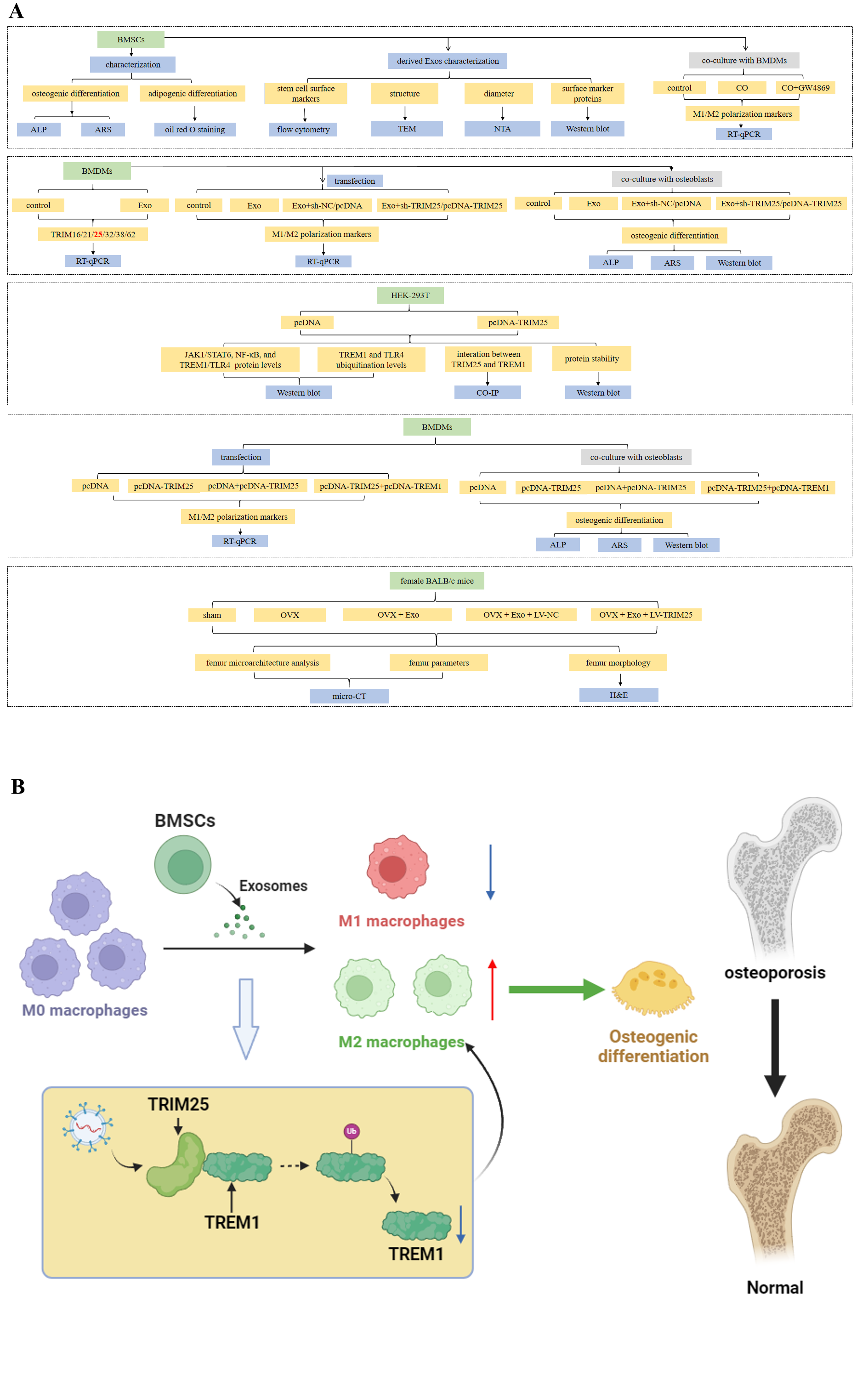

Supplement: Supplementary file 1 — Supplementary material 1. Mechanism idea diagram. A, Experimental flow chart; B, Experimental mechanism chart. [file 10020_2024_904_MOESM1_ESM.png]
